# Early Cardiac Workload and Long-Term Prognosis After Intracerebral Hemorrhage: Insights from a Large Multicenter Cohort

**DOI:** 10.31083/RCM49651

**Published:** 2026-06-17

**Authors:** Chuanying Wang, Yunyi Hao, Zeqiang Ji, Anxin Wang, Xiaoli Zhang, Yujie Zhou, Kaijiang Kang, Xingquan Zhao, Wenjuan Wang

**Affiliations:** ^1^Department of Neurology, Beijing Tiantan Hospital, Capital Medical University, 100070 Beijing, China; ^2^China National Clinical Research Center for Neurological Diseases, Beijing Tiantan Hospital, Capital Medical University, 100070 Beijing, China; ^3^Department of Clinical Epidemiology and Clinical Trial, Capital Medical University, 100054 Beijing, China; ^4^Department of Epidemiology, Beijing Neurosurgical Institute, Beijing Tiantan Hospital, Capital Medical University, 100070 Beijing, China; ^5^Department of Cardiology, Beijing Anzhen Hospital, Capital Medical University, 100029 Beijing, China; ^6^Research Unit of Artificial Intelligence in Cerebrovascular Disease, Chinese Academy of Medical Sciences, 100730 Beijing, China

**Keywords:** brain-heart interaction, cardiac workload, intracerebral hemorrhage, prognosis, rate-pressure product

## Abstract

**Background:**

Acute intracerebral hemorrhage (ICH) often induces a hyperadrenergic response, resulting in a significantly increased cardiac workload. This study aimed to assess the relationship between early cardiac workload and long-term outcomes following ICH, utilizing the rate-pressure product (RPP) as a simple surrogate indicator.

**Methods:**

We conducted an analysis of data from a large multicenter, prospective cohort comprising 1364 ICH patients. Heart rate (HR) and systolic blood pressure (SBP) were recorded to calculate the RPP. Multivariable logistic regression and Cox proportional hazards models were used to evaluate the associations of RPP with unfavorable functional outcome (modified Rankin Scale score >3) and all-cause mortality at 90 days and 1 year, respectively.

**Results:**

Elevated RPP was independently associated with unfavorable functional outcomes and all-cause mortality at 90 days and 1 year (all *p* < 0.05). RPP exhibited superior predictive performance, as indicated by higher C-statistics for all outcomes when compared to HR or SBP alone (all *p* < 0.05). A significant interaction was noted with in-hospital β-blocker treatment (*p* for interaction < 0.05), indicating that the association between high RPP and primary outcomes was attenuated in patients receiving β-blockers.

**Conclusions:**

Early cardiac workload, quantified by the RPP, is a potent independent predictor of long-term unfavorable functional outcome and all-cause mortality in patients with ICH.

## 1. Introduction

Intracerebral hemorrhage (ICH) is a devastating subtype of stroke accounting for 15–20% of all stroke cases, and is associated with significant mortality and long-term disability [1,2,3]. Recent evidence has increasingly underscored the critical role of the brain-heart interaction in the pathophysiology of acute brain injury [4,5,6]. Following the onset of ICH, neurohormonal and autonomic dysregulation frequently precipitate a hyperadrenergic state, which imposes substantial stress on the cardiovascular system [5,6].

This hyperadrenergic state triggers an acute increase in metabolic demand and hemodynamic burden, significantly elevating cardiac workload. When the cardiac workload surpasses the intrinsic cardiac reserve, the resulting mismatch between myocardial oxygen supply and consumption predisposes the myocardium to injury [7,8]. Furthermore, as optimal cardiac performance is crucial for maintaining cerebral perfusion pressure during acute brain injury, excessive cardiac workload may compromise the stability of cerebral blood flow [9,10], potentially exacerbating secondary brain injury and contributing to poor clinical outcomes. However, direct measurement of cardiac workload is technically complex [11,12] and challenging to implement during the acute phase of ICH. As a result, the clinical significance of early cardiac workload in patients with ICH remains inadequately elucidated.

The rate-pressure product (RPP), calculated as the product of heart rate (HR) and systolic blood pressure (SBP), serves as a well-validated and readily accessible surrogate for myocardial oxygen consumption [13,14]. It is widely utilized to quantify cardiac workload [15,16,17]. In the field of cardiology, RPP is a well-established prognostic marker for adverse outcomes in heart failure and coronary artery disease [18,19,20,21]. Additionally, among neurological disorders, RPP has been linked to mortality in acute aneurysmal subarachnoid hemorrhage and traumatic brain injury [15,22]. However, its prognostic significance in ICH has been rarely explored. Therefore, this study aimed to investigate the association between early cardiac workload, as measured by RPP, and long-term outcomes.

## 2. Methods

### 2.1 Study Design and Population

This study utilized data from a multicenter, prospective, consecutive, observational cohort that enrolled patients with ICH from 13 hospitals in Beijing between January 2014 and September 2016. The detailed design of this cohort has been previously described [23]. The cohort included patients aged 18 years or older who arrived at the hospital within 72 hours of symptom onset. Exclusion criteria encompassed a history of ICH or severe comorbidities, including: (i) Child-Pugh class C hepatic failure; (ii) renal failure (estimated glomerular filtration rate <15 mL/min/1.73 m^2^); (iii) heart failure (left ventricular ejection fraction <40%); and (iv) malignancy with a life expectancy of less than 3 months. A total of 1964 eligible patients were enrolled in the cohort. For the current analysis, patients with incomplete admission SBP or HR data (n = 181) or lacking follow-up information (n = 386) were further excluded. Additionally, patients with atrial fibrillation (AF, n = 33) were excluded because the irregular rhythm associated with AF renders HR an unreliable measure for estimating cardiac workload. Ultimately, 1364 patients were included in the final analysis.

### 2.2 Clinical Data Collection

Standardized questionnaires were employed to gather comprehensive clinical information, encompassing demographics (age and sex), lifestyle factors (smoking and alcohol intake), medical history (hypertension, diabetes mellitus, dyslipidemia, ischemic stroke, and myocardial infarction), and in-hospital medications (antihypertensive, antidiabetic, and lipid-lowering agents). The pre-morbid functional status was evaluated using the modified Rankin Scale (mRS) by trained investigators. Neurological deficit severity was assessed at admission utilizing the Glasgow Coma Scale (GCS). Neuroimaging assessments were conducted based on the initial computed tomography scan obtained within 24 hours of admission to ascertain hematoma characteristics, including location (lobar, deep, infratentorial, and ventricular), volume, and the presence of intraventricular extension.

### 2.3 RPP Calculation

Early cardiac workload was quantified using the RPP measured at admission within 72 hours of symptom onset (the cohort’s enrollment window), calculated as: [HR (bpm) × SBP (mmHg)] / 1000 [20]. Both SBP and HR were measured after a 5-minute rest period in a quiet environment, prior to the administration of any in-hospital medications. SBP was assessed using a mercury manometer while the patient was either seated or supine, and HR was derived from a 10-second, 12-lead electrocardiogram.

### 2.4 Follow-Up and Clinical Outcomes

Patients were followed up through telephone interviews at 90 days and 1 year after the onset of symptoms to evaluate their mRS scores. The primary outcomes included unfavorable functional outcome, defined as an mRS score of >3, and all-cause mortality. All outcome assessors were blinded to the patients’ baseline clinical and imaging data.

### 2.5 Statistical Analysis

Continuous variables exhibiting non-normal distribution were presented as medians with interquartile ranges (IQRs), whereas categorical variables were displayed as counts and percentages. The Kruskal-Wallis test and the chi-square test (or Fisher’s exact test when appropriate) were employed to compare baseline characteristics across the quartiles of RPP (Q1, Q2, Q3, and Q4).

Associations of RPP with unfavorable functional outcomes and all-cause mortality were evaluated using multivariable logistic regression and Cox proportional hazards models, respectively. RPP was modeled both continuously and categorically by quartiles. Four sequential models were fitted: Model 1, unadjusted; Model 2, adjusted for age and sex; Model 3, additionally adjusted for smoking, alcohol intake, medical history (hypertension, diabetes mellitus, dyslipidemia, ischemic stroke, myocardial infarction), mRS before onset, admission GCS, location of hematoma, hematoma volume, and intraventricular extension; Model 4, further adjusted for in-hospital medications (antihypertensive, antidiabetic, and lipid-lowering agents). Trend test was used to assess the impact of RPP elevation across quartiles on outcomes. To further assess the dose-response relationship, restricted cubic splines (RCS) based on Model 4 were employed, with 3–5 knots selected according to the Akaike Information Criterion and placed at prespecified percentiles. Shift analysis of the full mRS distribution at 90 days and 1 year across RPP quartiles was conducted using ordinal logistic regression and the Kruskal–Wallis test. One-year survival across RPP quartiles was compared using Kaplan-Meier curves and the log-rank test. The predictive ability of RPP, HR, and SBP was compared using C-statistics.

To assess the robustness of the observed associations, sensitivity analyses were performed by restricting the cohort to patients with onset-to-door times of ≤6 h, ≤12 h, and ≤24 h. Furthermore, subgroup analyses were conducted to assess the consistency of the findings across different populations: age (<65 years vs. ≥65 years), sex, history of hypertension, onset-to-door time (≤6 h, 6–12 h, and >12 h), ICH severity (GCS >12, 9–12, and ≤8) and in-hospital β-blocker treatment.

All statistical analyses were performed using R software (version 4.4.2; R Foundation for Statistical Computing, Vienna, Austria), and a two-sided *p*-value < 0.05 was considered statistically significant.

## 3. Results

### 3.1 Baseline Characteristics

A total of 1364 patients with ICH were included in this study. The median age was 57.0 years (IQR, 48.0–67.0), and 915 (67.1%) patients were male. Baseline characteristics according to quartiles of RPP are detailed in Table 1. Significant differences were observed among the quartiles concerning history of hypertension, history of diabetes mellitus, onset-to-door time, admission SBP, admission HR, admission GCS score, location of hematoma, hematoma volume, intraventricular extension, and in-hospital antihypertensive agents (all *p* < 0.05).

**Table 1.** **Baseline characteristics among ICH patients according to quartiles of RPP**.

Variables	Total	RPP				*p*-value
		Q1 (<10.89)	Q2 (10.89–12.88)	Q3 (12.88–15.33)	Q4 (>15.33)	
n	1364	341	341	341	341	
Age, years	57.0 (48.0, 67.0)	59.0 (47.0, 67.0)	58.0 (50.0, 67.0)	57.0 (50.0, 65.0)	56.0 (47.0, 66.0)	0.448
Male, n (%)	915 (67.1)	230 (67.4)	224 (65.7)	230 (67.4)	231 (67.7)	0.939
Smoking, n (%)	585 (42.9)	151 (44.3)	144 (42.2)	152 (44.6)	138 (40.5)	0.673
Alcohol intake, n (%)	499 (36.6)	128 (37.5)	129 (37.8)	122 (35.8)	120 (35.2)	0.863
Medical history, n (%)						
Hypertension	934 (68.5)	170 (49.9)	234 (68.6)	258 (75.7)	272 (79.8)	<0.001
Diabetes mellitus	205 (15.0)	39 (11.4)	46 (13.5)	49 (14.4)	71 (20.8)	0.004
Dyslipidemia	125 (9.2)	32 (9.4)	32 (9.4)	32 (9.4)	29 (8.5)	0.971
Ischemic stroke	195 (14.3)	52 (15.2)	46 (13.5)	46 (13.5)	51 (15.0)	0.865
Myocardial infarction	28 (2.1)	9 (2.6)	8 (2.3)	7 (2.1)	4 (1.2)	0.564
mRS before onset	0.0 (0.0, 0.0)	0.0 (0.0, 0.0)	0.0 (0.0, 0.0)	0.0 (0.0, 0.0)	0.0 (0.0, 0.0)	0.926
Onset-to-door time, h	4.0 (1.9, 11.4)	5.5 (2.3, 19.0)	4.5 (2.0, 11.4)	3.0 (1.7, 8.4)	3.5 (1.8, 8.4)	<0.001
Admission SBP, mmHg	164.0 (147.0, 185.3)	135.0 (127.0, 150.0)	159.0 (148.0, 170.0)	177.0 (162.0, 188.0)	195.0 (175.0, 210.0)	<0.001
Admission HR, bpm	79.0 (70.0, 88.0)	70.0 (62.0, 75.0)	76.0 (70.0, 80.0)	80.0 (75.0, 86.0)	98.0 (86.0, 108.0)	<0.001
Admission GCS score	14.0 (9.0, 15.0)	14.0 (12.0, 15.0)	14.0 (11.0, 15.0)	14.0 (10.0, 15.0)	10.0 (5.0, 14.0)	<0.001
Location of hematoma, n (%)						0.033
Lobar	351 (25.7)	106 (31.1)	90 (26.4)	84 (24.6)	71 (20.8)	
Deep	772 (56.6)	180 (52.8)	195 (57.2)	206 (60.4)	191 (56.0)	
Infratentorial	155 (11.4)	33 (9.7)	36 (10.6)	36 (10.6)	50 (14.7)	
Ventricular	86 (6.3)	22 (6.5)	20 (5.9)	15 (4.4)	29 (8.5)	
Hematoma volume, mL	17.1 (6.7, 37.0)	15.3 (5.2, 30.2)	14.0 (5.0, 27.9)	15.4 (7.0, 37.1)	27.0 (10.0, 49.5)	<0.001
Intraventricular extension, n (%)	488 (35.8)	99 (29.0)	100 (29.3)	120 (35.2)	169 (49.6)	<0.001
In-hospital medications, n (%)						
Antihypertensive agents	1049 (76.9)	193 (56.6)	267 (78.3)	299 (87.7)	290 (85.0)	<0.001
β-blocker	143 (10.5)	20 (5.9)	32 (9.4)	47 (13.8)	44 (12.9)	0.003
Antidiabetic agents	94 (6.9)	26 (7.6)	25 (7.3)	29 (8.5)	14 (4.1)	0.117
Lipid-lowering agents	196 (14.4)	52 (15.2)	41 (12.0)	48 (14.1)	55 (16.1)	0.454

Note: Non-normal continuous data were expressed as median (interquartile range) and categorical variables were described as n (%). Abbreviations: GCS, Glasgow Coma Scale; HR, heart rate; ICH, intracerebral hemorrhage; mRS, modified Rankin Scale; RPP, rate-pressure product; SBP, systolic blood pressure.

### 3.2 Association Between RPP and Primary Outcomes

At the 90-day follow-up, unfavorable functional outcome was observed in 560 patients (41.1%), and 281 patients (20.6%) had died. By one year, 466 patients (34.2%) experienced an unfavorable functional outcome, while 334 patients (24.5%) had succumbed to their condition.

**Fig. 1.**
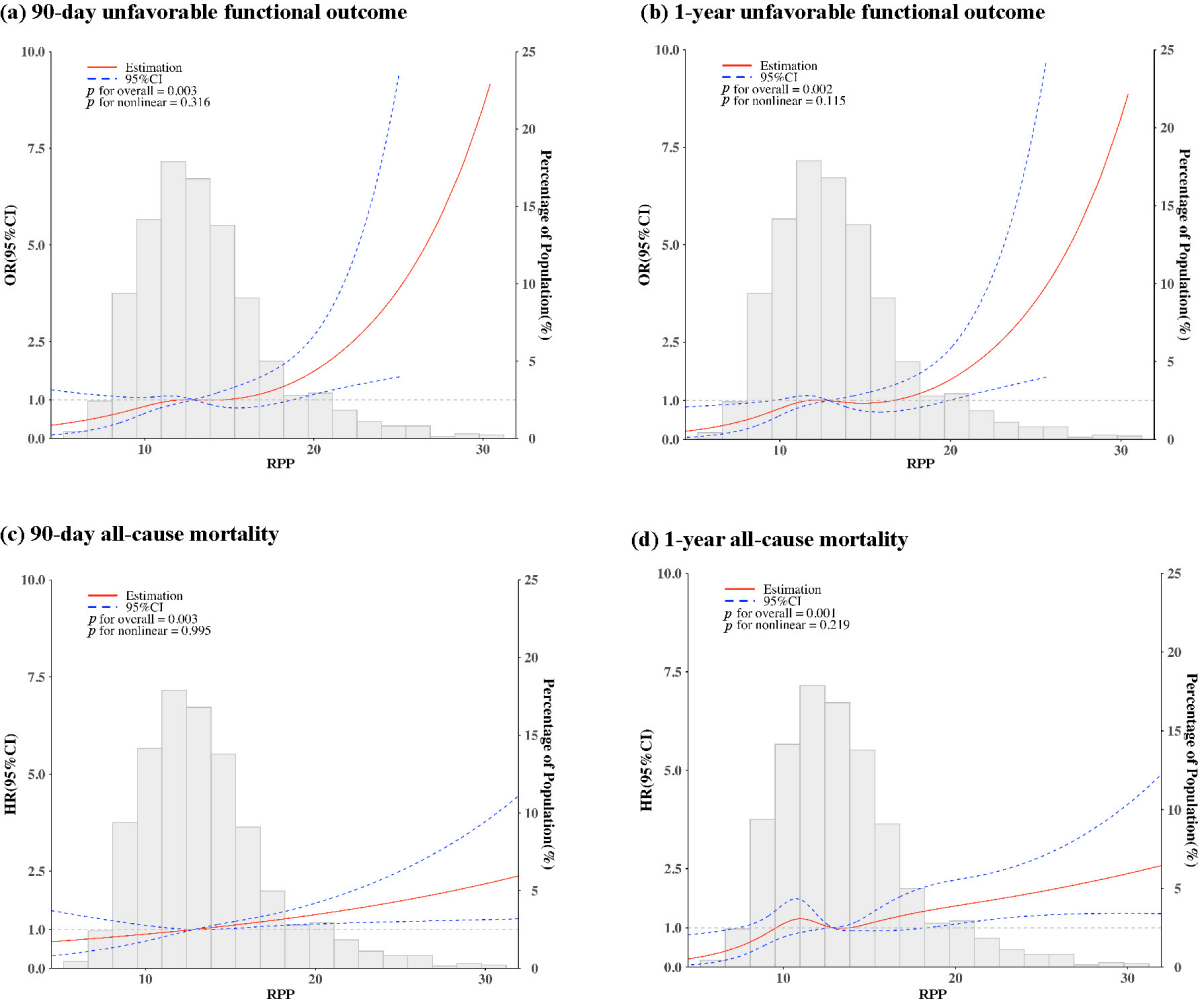
**Restricted cubic spline curves of the association between rate-pressure product (RPP) and primary outcomes**. The red solid lines represent the odds ratios (ORs) for (a) 90-day unfavorable functional outcome and (b) 1-year unfavorable functional outcome, and the hazard ratios (HRs) for (c) 90-day all-cause mortality and (d) 1-year all-cause mortality. The blue dashed lines indicate the 95% confidence intervals (CIs), and the gray bars represent histograms of RPP distribution. Models were adjusted for age, sex, smoking, alcohol intake, medical history (hypertension, diabetes mellitus, dyslipidemia, ischemic stroke, myocardial infarction), modified Rankin Scale score before onset, admission Glasgow Coma Scale, location of hematoma, hematoma volume, intraventricular extension and in-hospital medications (antihypertensive, antidiabetic, and lipid-lowering agents).

When RPP was analyzed as a continuous variable, each 1-unit increase was independently associated with higher odds of primary outcomes across all models (Table 2). In the fully adjusted Model 4, the odds ratio (OR) was 1.084 (95% confidence interval [CI]: 1.036–1.133) for 90-day unfavorable functional outcome, 1.082 (95% CI: 1.035–1.132) for 1-year unfavorable functional outcome; the hazard ratio (HR) was 1.046 (95% CI: 1.019–1.074) for 90-day all-cause mortality and 1.050 (95% CI: 1.024–1.076) for 1-year all-cause mortality. Furthermore, the RCS curves illustrated a general monotonic increase in the risk of all outcomes with rising RPP levels (*p* for overall < 0.05 and *p* for nonlinear > 0.05 for all outcomes; Fig. 1).

**Table 2.** **Association between rate-pressure product (RPP) and primary outcomes**.

Outcomes	RPP as a continuous variable	RPP as a categorical variable, quartiles				
		Q1	Q2	Q3	Q4	*p* for trend
90-day unfavorable functional outcome, OR (95% CI)						
n (%)	560 (41.1)	101 (29.6)	117 (34.3)	132 (38.7)	210 (61.6)	
Model 1	1.170 (1.134–1.208)	Reference	1.241 (0.899–1.715)	1.501 (1.092–2.067)	3.809 (2.777–5.255)	<0.001
Model 2	1.185 (1.147–1.225)	Reference	1.198 (0.860–1.672)	1.510 (1.088–2.101)	4.182 (3.009–5.852)	<0.001
Model 3	1.080 (1.034–1.128)	Reference	1.149 (0.755–1.752)	1.250 (0.817–1.916)	1.807 (1.160–2.821)	0.009
Model 4	1.084 (1.036–1.133)	Reference	1.190 (0.776–1.826)	1.303 (0.840–2.026)	1.851 (1.173–2.927)	0.008
1-year unfavorable functional outcome, OR (95% CI)						
n (%)	466 (34.2)	83 (24.3)	95 (27.9)	111 (32.6)	177 (51.9)	
Model 1	1.159 (1.124–1.195)	Reference	1.200 (0.853–1.692)	1.500 (1.074–2.102)	3.355 (2.428–4.664)	<0.001
Model 2	1.181 (1.143–1.221)	Reference	1.163 (0.816–1.661)	1.536 (1.085–2.181)	3.790 (2.696–5.368)	<0.001
Model 3	1.074 (1.029–1.122)	Reference	1.142 (0.732–1.786)	1.380 (0.881–2.167)	1.591 (1.003–2.529)	0.033
Model 4	1.082 (1.035–1.132)	Reference	1.224 (0.779–1.928)	1.531 (0.964–2.441)	1.709 (1.065–2.750)	0.017
90-day all-cause mortality, HR (95% CI)						
n (%)	281 (20.6)	43 (12.6)	51 (15.0)	60 (17.6)	127 (37.2)	
Model 1	1.155 (1.128–1.182)	Reference	1.177 (0.783–1.769)	1.440 (0.973–2.130)	3.506 (2.480–4.957)	<0.001
Model 2	1.160 (1.133–1.188)	Reference	1.159 (0.770–1.742)	1.439 (0.973–2.130)	3.538 (2.502–5.003)	<0.001
Model 3	1.041 (1.014–1.068)	Reference	1.059 (0.696–1.612)	1.104 (0.732–1.665)	1.384 (0.947–2.022)	<0.001
Model 4	1.046 (1.019–1.074)	Reference	1.075 (0.705–1.639)	1.212 (0.798–1.840)	1.526 (1.040–2.240)	<0.001
1-year all-cause mortality, HR (95% CI)						
n (%)	334 (24.5)	57 (16.7)	64 (18.8)	71 (20.8)	142 (41.6)	
Model 1	1.144 (1.119–1.170)	Reference	1.147 (0.803–1.639)	1.296 (0.914–1.836)	3.064 (2.253–4.167)	<0.001
Model 2	1.153 (1.127–1.180)	Reference	1.126 (0.788–1.610)	1.305 (0.921–1.850)	3.113 (2.288–4.237)	<0.001
Model 3	1.045 (1.019–1.071)	Reference	1.059 (0.734–1.530)	1.086 (0.753–1.565)	1.391 (0.991–1.953)	<0.001
Model 4	1.050 (1.024–1.076)	Reference	1.092 (0.756–1.577)	1.209 (0.834–1.753)	1.539 (1.093–2.168)	<0.001

Model 1: Unadjusted; Model 2: Adjusted for age and sex; Model 3: Adjusted for variables in Model 2 plus smoking, alcohol intake, medical history (hypertension, diabetes mellitus, dyslipidemia, ischemic stroke, myocardial infarction), modified Rankin Scale score before onset, admission Glasgow Coma Scale, location of hematoma, hematoma volume, intraventricular extension. Model 4: Adjusted for variables in Model 3 plus in-hospital medications (antihypertensive, antidiabetic, and lipid-lowering agents). Abbreviations: CI, confidence interval; HR, hazard ratio; OR, odds ratio.

**Fig. 2.**
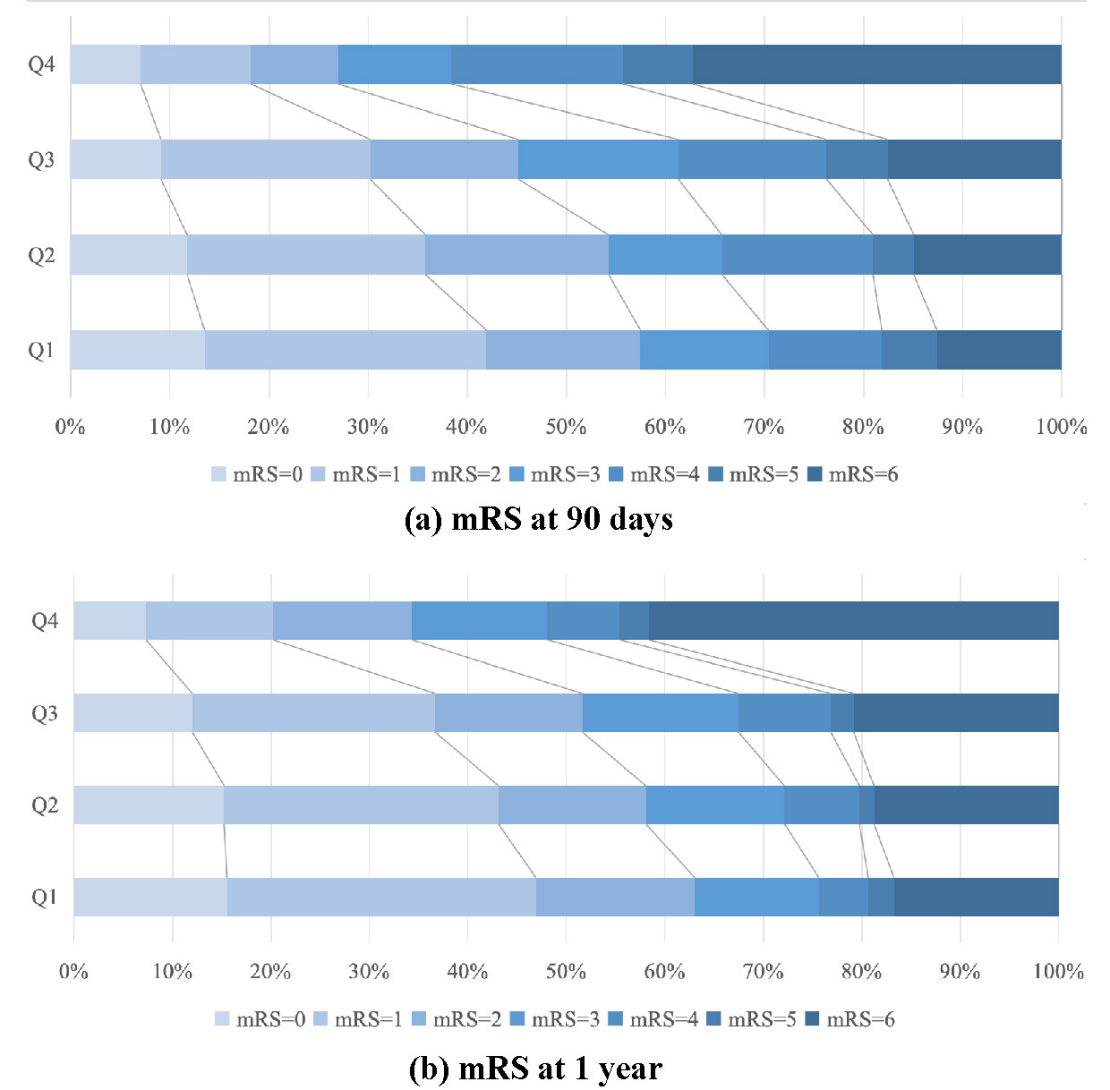
**Distribution of modified Rankin Scale (mRS) scores across quartiles of rate-pressure product**. (a) mRS scores at 90 days. (b) mRS scores at 1 year.

**Fig. 3.**
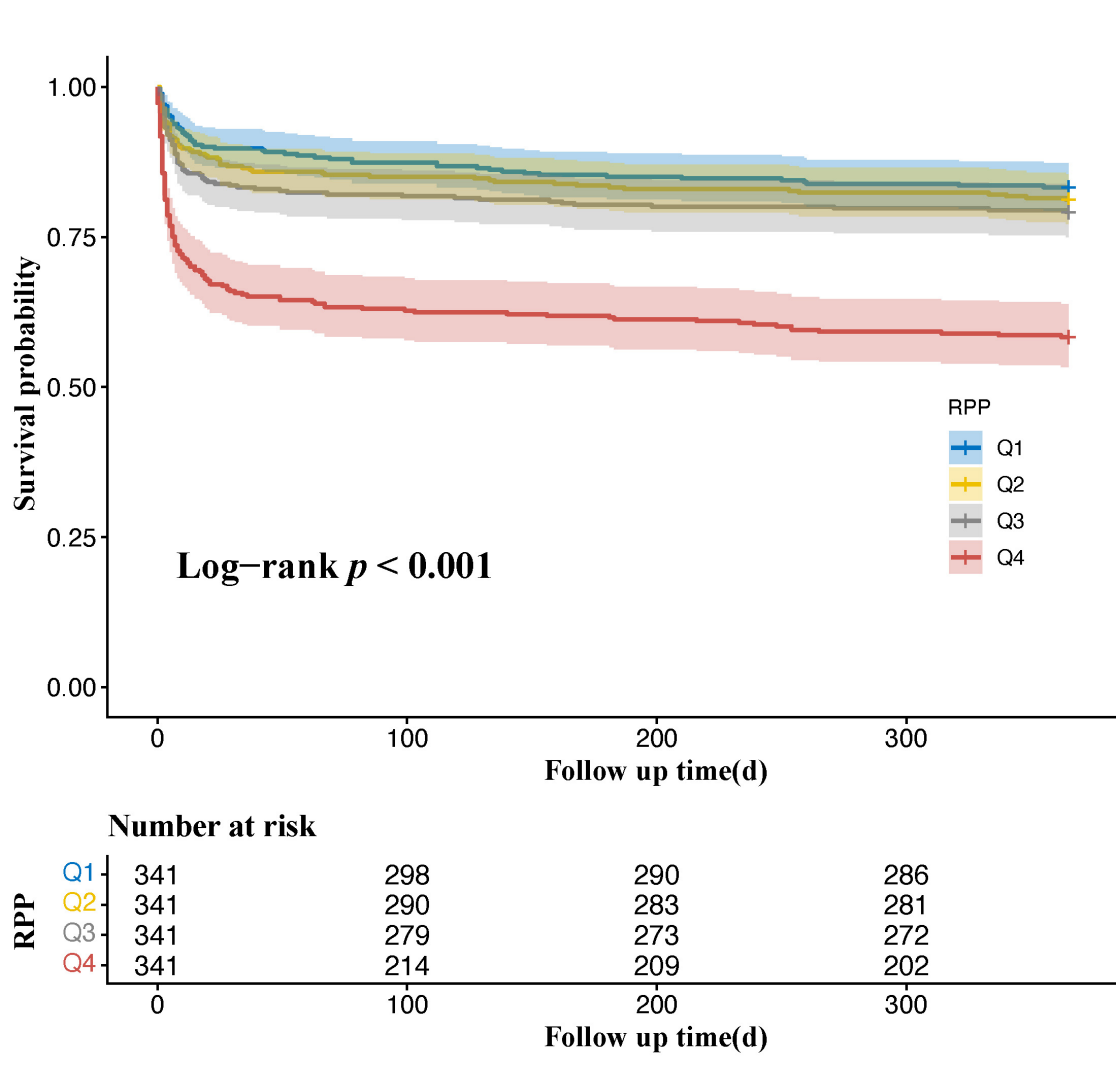
**1-year Kaplan-Meier survival curves across quartiles of rate-pressure product**.

When RPP was categorized into quartiles, patients in the highest quartile (Q4) exhibited a significantly higher risk of primary outcomes compared to those in the lowest quartile (Q1) (Table 2). In Model 4, the multivariable-adjusted OR for Q4 versus Q1 was 1.851 (95% CI: 1.173–2.927) for 90-day unfavorable functional outcome and 1.709 (95% CI: 1.065–2.750) for 1-year unfavorable functional outcome; adjusted HR was 1.526 (95% CI: 1.040–2.240) for 90-day all-cause mortality and 1.539 (95% CI: 1.093–2.168) for 1-year all-cause mortality. Trend analyses revealed a statistically significant association between increasing RPP quartiles and primary outcomes (Table 2; *p* for trend < 0.05 for all). Furthermore, shift analysis showed that as RPP quartiles increased, the distribution of mRS scores exhibited a significant shift toward worse functional outcome at both 90 days and 1 year (ordinal logistic regression overall LR χ^2^ = 99.851 and 88.063, respectively; Kruskal–Wallis H = 96.135 and 86.369, respectively; all *p* < 0.001; Fig. 2). Kaplan-Meier survival curves demonstrated a significant difference in one-year survival rates across RPP quartiles (log-rank χ^2 ^= 86.32, df = 3, *p* < 0.001). Specifically, the lowest cumulative survival probability was observed in the Q4 group (Fig. 3).

### 3.3 Sensitivity Analysis

The results of sensitivity analysis are shown in **Supplementary Table 1**. The relationship between RPP and outcomes remained robust across different time windows: ≤6 h (n = 838), ≤12 h (n = 1058), and ≤24 h (n = 1189). In the fully adjusted model, elevated RPP was consistently associated with a higher risk of unfavorable functional outcomes and mortality.

### 3.4 Subgroup Analysis

**Fig. 4.**
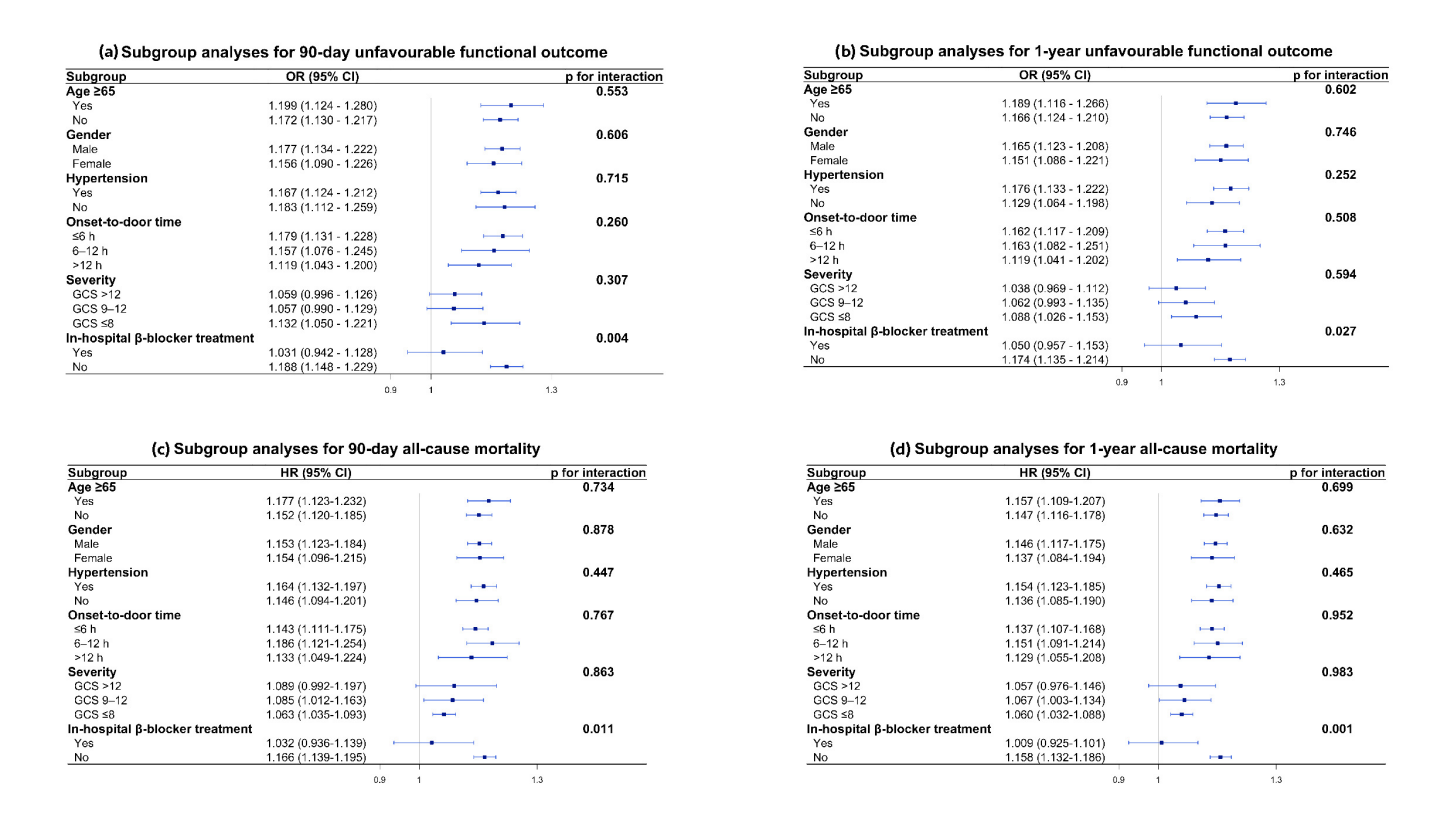
**Subgroup analyses of the association between rate-pressure product and outcomes**. Forest plots show (a) 90-day unfavorable functional outcome (OR), (b) 1-year unfavorable functional outcome (OR), (c) 90-day all-cause mortality (HR), and (d) 1-year all-cause mortality (HR) across subgroups. Abbreviations: CI, confidence interval; GCS, Glasgow Coma Scale; HR, hazard ratio; OR, odds ratio.

The association between RPP and all outcomes remained consistent across subgroups stratified by age, sex, history of hypertension, onset-to-door time and ICH severity, with no significant interactions observed (*p* for interaction > 0.05 for all). However, a significant interaction was detected with in-hospital β-blocker treatment (*p* for interaction < 0.05 for all four outcomes). The association between high RPP and primary outcomes was attenuated in patients receiving β-blockers (Fig. 4).

### 3.5 Predictive Performance of RPP

The predictive performance of the RPP was evaluated against its individual components, HR and SBP, utilizing C-statistics (Table 3). The C-statistic of RPP was 0.643 (95% CI, 0.613–0.673) for 90-day unfavorable functional outcome, 0.637 (95% CI, 0.606–0.669) for 1-year unfavorable functional outcome, 0.668 (95% CI, 0.630–0.706) for 90-day all-cause mortality, and 0.650 (95% CI, 0.614–0.686) for 1-year all-cause mortality. When compared to HR or SBP alone, RPP demonstrated a significantly superior predictive capability for all four outcomes (*p* < 0.05 for all comparisons).

**Table 3.** **Comparison of the predictive performance of RPP, HR and SBP for outcomes in patients with intracerebral hemorrhage**.

Outcomes	C-statistic (95% CI)			*p*-value	
	RPP	HR	SBP	RPP vs. HR	RPP vs. SBP
90-day unfavorable functional outcome	0.643 (0.613–0.673)	0.614 (0.583–0.645)	0.604 (0.574–0.635)	0.015	0.001
1-year unfavorable functional outcome	0.637 (0.606–0.669)	0.612 (0.578–0.645)	0.597 (0.565–0.629)	0.035	0.001
90-day all-cause mortality	0.668 (0.630–0.706)	0.638 (0.597–0.678)	0.623 (0.584–0.662)	0.039	0.003
1-year all-cause mortality	0.650 (0.614–0.686)	0.619 (0.581–0.656)	0.611 (0.575–0.647)	0.023	0.006

Abbreviations: CI, confidence interval; HR, heart rate; RPP, Rate-pressure product; SBP, systolic blood pressure.

## 4. Discussion

In this large, multicenter, prospective cohort study, we systematically demonstrated that (1) RPP was independently associated with long-term unfavorable functional outcome and all-cause mortality in patients with acute ICH; (2) RPP exhibited greater predictive performance compared with either HR or SBP alone; and (3) the adverse prognostic risk associated with high RPP was mitigated by in-hospital β-blocker treatment. Collectively, these findings identify elevated early cardiac workload as a potent prognostic factor for poor long-term outcomes following ICH.

Although the precise mechanisms linking cardiac workload to ICH prognosis remain incompletely understood, we postulate that the pathological activation of the brain-heart axis plays a pivotal role. First, the mass effect of the hematoma and surrounding edema, along with the toxic effects of blood breakdown products, may structurally and functionally compromise the central autonomic network (CAN) [24]. This injury can lead to the disinhibition of sympatho-excitatory centers, precipitating a hyperadrenergic state. Such sympathetic hyperactivity has been shown to aggravate secondary brain injury by inducing disruption of the blood-brain barrier, exacerbating perihematomal edema, and promoting a pro-inflammatory state [24,25,26], ultimately worsening patient prognosis [27,28]. Second, previous studies have demonstrated that stroke events can trigger neurovascular uncoupling and impair cerebral autoregulation, making cerebral blood flow more dependent on systemic hemodynamics [6,29]. In this vulnerable state, elevated cardiac workload may not only impair cerebral perfusion but also transmit excessive hydrostatic pressure to the injured neurovascular unit, potentially exacerbating perihematomal edema and increasing the risk of hematoma expansion, thereby worsening neurological injury. Third, the RPP, also known as the double product, is a well-validated, non-invasive index of myocardial oxygen consumption [13,14]. During exercise and stress conditions, myocardial oxygen demand rises largely in proportion to the tension-time work and afterload faced by the left ventricle; HR strongly determines the frequency and cumulative duration of systolic tension development, whereas SBP serves as a convenient clinical surrogate for left ventricular afterload [30,31]. Accordingly, RPP increases in parallel with oxygen requirements. Classic invasive studies in patients with angina demonstrated a close relationship between RPP and directly assessed indices of myocardial oxygen consumption and myocardial blood flow [13,32]. Consequently, a high admission RPP may indicate an abrupt surge in myocardial oxygen demand. This acute demand-supply mismatch may deplete cardiac reserve and trigger subclinical myocardial injury [7,8], thereby reducing physiological resilience to post-ICH complications.

Our findings align with a previous smaller retrospective study that identified RPP as a predictor of short-term (30-day) mortality [33]. However, our study further extends prior evidence by demonstrating associations with long-term unfavorable functional outcomes and mortality. Importantly, these associations remain significant even after adjusting for baseline severity markers such as GCS and hematoma volume. This finding implies that elevated RPP is not merely an epiphenomenon of the initial injury severity. Instead, it likely reflects a component of systemic physiological dysregulation that is independently associated with poor functional recovery.

Furthermore, we found that RPP demonstrated superior prognostic performance to either HR or SBP alone. This observation aligns with existing cardiology literature regarding coronary artery disease [21,34]. Mechanistically, while SBP primarily reflects left ventricular afterload and HR reflects the chronotropic state, RPP integrates both parameters, providing a more comprehensive perspective on cardiac work. Furthermore, the physiological relationship between BP and HR is precisely regulated by autonomic mechanisms, such as the baroreceptor reflex, which maintain a dynamic negative feedback balance in a healthy state [35]. However, in acute ICH, CAN injury can disrupt this autonomic feedback [36,37], resulting in a simultaneous elevation of SBP and HR. RPP effectively captures this “double hit” on the cardiovascular system, thus providing a more accurate reflection of the severity of systemic physiological dysregulation.

A critical consideration in utilizing admission physiological parameters is the timing of measurement. While physiological changes are most volatile in the first few hours, accumulating evidence suggests that autonomic dysregulation after ICH is a sustained pathological state that persists throughout the early acute phase [38]. Sykora et al. demonstrated that baroreflex sensitivity remains significantly impaired in ICH patients within 72 hours of symptom onset compared to healthy controls [36]. Therefore, the RPP obtained within this window may reasonably capture the pathophysiology of autonomic dysregulation after ICH. Importantly, our sensitivity and subgroup analyses revealed that the association between RPP and outcomes remained consistent across all time windows, suggesting that the prognostic signal of RPP is not driven by a specific arrival window. Collectively, these findings establish admission RPP as a temporally stable and reliable prognostic biomarker during the early phase of ICH.

Current evidence regarding the use of β-blockers in ICH remains inconsistent. While some studies suggested benefits, including reduced perihematomal edema, lower infection rates, and decreased mortality [39,40,41], a recent large meta-analysis found no significant association with functional outcome or survival [42]. These divergent findings are likely attributable to the profound clinical and physiological heterogeneity of the ICH population, highlighting the need for a more targeted therapeutic strategy. Our study proposes a potential direction for stratification. We observed that the association between high RPP and poor outcomes was attenuated in patients receiving β-blockers. Given that β-blockers exert sympatholytic effects, this finding supports the hypothesis that high RPP may identify a hyperadrenergic phenotype. Therefore, RPP holds the potential to serve as a biomarker for stratifying patients who might derive greater benefit from targeted sympatholytic therapy. This hypothesis warrants prospective validation in randomized controlled trials.

While the brain-heart interaction is well-documented in the context of ischemic stroke, data regarding ICH remain limited [4,43]. Existing studies on ICH have predominantly focused on overt cardiac pathologies, such as myocardial infarction, heart failure, and severe arrhythmias [44,45,46], neglecting the clinical significance of concealed cardiovascular stress. Our findings underscore the prognostic value of early cardiac workload assessment using the RPP, an easily obtainable indicator. Incorporating RPP into initial risk stratification may assist clinicians in identifying patients experiencing excessive cardiovascular stress who may require intensified hemodynamic monitoring beyond standard blood pressure management.

### Limitations

Several limitations must be acknowledged. First, as this is an observational study, the potential for residual confounding cannot be entirely ruled out, despite comprehensive multivariable adjustment. Second, due to limitations in the database, cardiac workload was assessed using a single admission RPP measurement, which may not fully reflect the dynamic nature of sympathetic activation and hemodynamic fluctuations during the early phase of ICH. The trajectory of RPP over the first 24–72 hours may hold greater prognostic value than the initial baseline value alone. Future studies utilizing continuous hemodynamic monitoring or repeated-measures analysis are warranted to better capture the impact of these dynamic fluctuations on clinical outcomes. Third, we lacked specific data regarding the cause of death (cardiac vs. neurological), as well as access to cardiac imaging or biomarker data to definitively confirm cardiac injury. Fourth, our cohort was predominantly male (67%), which may limit the generalizability of the findings to female patients. Future studies with a more balanced sex distribution are warranted to validate the present results.

## 5. Conclusions

In this large, multicenter, prospective cohort (67.1% male), early cardiac workload, quantified by the RPP, serves as a potent independent predictor of long-term unfavorable functional outcome and all-cause mortality in patients with ICH. These findings underscore the prognostic relevance of the brain–heart interaction and highlight the potential clinical value of monitoring cardiovascular stress during the acute phase of ICH.

## Data Availability

The datasets used during the current study are available from the corresponding author on reasonable request.

## References

[1] Sheth KN (2022). Spontaneous Intracerebral Hemorrhage. The New England Journal of Medicine.

[2] van Asch CJ, Luitse MJ, Rinkel GJ, van der Tweel I, Algra A, Klijn CJ (2010). Incidence, case fatality, and functional outcome of intracerebral haemorrhage over time, according to age, sex, and ethnic origin: a systematic review and meta-analysis. The Lancet. Neurology.

[3] An SJ, Kim TJ, Yoon BW (2017). Epidemiology, Risk Factors, and Clinical Features of Intracerebral Hemorrhage: An Update. Journal of Stroke.

[4] Scheitz JF, Sposato LA, Schulz-Menger J, Nolte CH, Backs J, Endres M (2022). Stroke-Heart Syndrome: Recent Advances and Challenges. Journal of the American Heart Association.

[5] Sposato LA, Hilz MJ, Aspberg S, Murthy SB, Bahit MC, Hsieh CY (2020). Post-Stroke Cardiovascular Complications and Neurogenic Cardiac Injury: JACC State-of-the-Art Review. Journal of the American College of Cardiology.

[6] Chen Z, Venkat P, Seyfried D, Chopp M, Yan T, Chen J (2017). Brain-Heart Interaction: Cardiac Complications After Stroke. Circulation Research.

[7] DeFilippis AP, Chapman AR, Mills NL, de Lemos JA, Arbab-Zadeh A, Newby LK (2019). Assessment and Treatment of Patients With Type 2 Myocardial Infarction and Acute Nonischemic Myocardial Injury. Circulation.

[8] Deedwania PC, Carbajal EV (1992). Role of myocardial oxygen demand in the pathogenesis of silent ischemia during daily life. The American Journal of Cardiology.

[9] Meng L, Hou W, Chui J, Han R, Gelb AW (2015). Cardiac Output and Cerebral Blood Flow: The Integrated Regulation of Brain Perfusion in Adult Humans. Anesthesiology.

[10] Krishnamoorthy V, Mackensen GB, Gibbons EF, Vavilala MS (2016). Cardiac Dysfunction After Neurologic Injury: What Do We Know and Where Are We Going?. Chest.

[11] Takaoka H, Takeuchi M, Odake M, Yokoyama M (1992). Assessment of myocardial oxygen consumption (Vo2) and systolic pressure-volume area (PVA) in human hearts. European Heart Journal.

[12] Papadopoulos K, Özden Tok Ö, Mitrousi K, Ikonomidis I (2021). Myocardial Work: Methodology and Clinical Applications. Diagnostics (Basel, Switzerland).

[13] Gobel FL, Norstrom LA, Nelson RR, Jorgensen CR, Wang Y (1978). The rate-pressure product as an index of myocardial oxygen consumption during exercise in patients with angina pectoris. Circulation.

[14] Nelson RR, Gobel FL, Jorgensen CR, Wang K, Wang Y, Taylor HL (1974). Hemodynamic predictors of myocardial oxygen consumption during static and dynamic exercise. Circulation.

[15] Krishnamoorthy V, Vavilala MS, Chaikittisilpa N, Rivara FP, Temkin NR, Lele AV (2018). Association of Early Myocardial Workload and Mortality Following Severe Traumatic Brain Injury. Critical Care Medicine.

[16] Whitman M, Jenkins C, Sabapathy S, Adams L (2019). Comparison of Heart Rate Blood Pressure Product Versus Age-Predicted Maximum Heart Rate as Predictors of Cardiovascular Events During Exercise Stress Echocardiography. The American Journal of Cardiology.

[17] Bassingthwaighte JB, Beard DA, Carlson BE, Dash RK, Vinnakota K (2012). Modeling to link regional myocardial work, metabolism and blood flows. Annals of Biomedical Engineering.

[18] White WB (1999). Heart rate and the rate-pressure product as determinants of cardiovascular risk in patients with hypertension. American Journal of Hypertension.

[19] Verma AK, Sun JL, Hernandez A, Teerlink JR, Schulte PJ, Ezekowitz J (2018). Rate pressure product and the components of heart rate and systolic blood pressure in hospitalized heart failure patients with preserved ejection fraction: Insights from ASCEND-HF. Clinical Cardiology.

[20] Zhou J, Li YJ, Zhou XD, Wang LJ (2024). Rate-Pressure Product is a Novel Predictor for Short- and Long-Term Mortality in Patients with Acute Coronary Syndrome Undergoing Primary PCI/Immediate Invasive Strategy. Clinical Interventions in Aging.

[21] Yazdani B, Kleber ME, Yücel G, Delgado GE, Benck U, Krüger B (2020). Association of double product and pulse pressure with cardiovascular and all-cause mortality in the LURIC study. Journal of Clinical Hypertension (Greenwich, Conn.).

[22] Zhao J, Zhang S, Ma J, Shi G, Zhou J (2022). Admission rate-pressure product as an early predictor for in-hospital mortality after aneurysmal subarachnoid hemorrhage. Neurosurgical Review.

[23] Wang C, Wang W, Li G, Wang A, Zhang X, Xiong Y (2022). Prognostic value of glycemic gap in patients with spontaneous intracerebral hemorrhage. European Journal of Neurology.

[24] Kang K, Shi K, Liu J, Li N, Wu J, Zhao X (2024). Autonomic dysfunction and treatment strategies in intracerebral hemorrhage. CNS Neuroscience & Therapeutics.

[25] Sykora M, Diedler J, Turcani P, Rupp A, Steiner T (2009). Subacute perihematomal edema in intracerebral hemorrhage is associated with impaired blood pressure regulation. Journal of the Neurological Sciences.

[26] Demura M, Ishii H, Takarada-Iemata M, Kamide T, Yoshikawa A, Nakada M (2023). Sympathetic Nervous Hyperactivity Impairs Microcirculation Leading to Early Brain Injury After Subarachnoid Hemorrhage. Stroke.

[27] Tschoe C, Bushnell CD, Duncan PW, Alexander-Miller MA, Wolfe SQ (2020). Neuroinflammation after Intracerebral Hemorrhage and Potential Therapeutic Targets. Journal of Stroke.

[28] Bautista W, Adelson PD, Bicher N, Themistocleous M, Tsivgoulis G, Chang JJ (2021). Secondary mechanisms of injury and viable pathophysiological targets in intracerebral hemorrhage. Therapeutic Advances in Neurological Disorders.

[29] Tranmer BI, Keller TS, Kindt GW, Archer D (1992). Loss of cerebral regulation during cardiac output variations in focal cerebral ischemia. Journal of Neurosurgery.

[30] Fletcher GF, Balady GJ, Amsterdam EA, Chaitman B, Eckel R, Fleg J (2001). Exercise standards for testing and training: a statement for healthcare professionals from the American Heart Association. Circulation.

[31] Sarnoff SJ, Braunwald E, Welch GH, Case RB, Stainsby WN, Macruz R (1958). Hemodynamic determinants of oxygen consumption of the heart with special reference to the tension-time index. The American Journal of Physiology.

[32] Jorgensen CR, Gobel FL, Taylor HL, Wang Y (1977). Myocardial blood flow and oxygen consumption during exercise. Annals of the New York Academy of Sciences.

[33] Zheng H, Tang Y, Zhou H, Ji X (2024). The rate-pressure product combined model within 24 h on admission predicts the 30-day mortality rate in conservatively treated patients with intracerebral hemorrhage. Frontiers in Neurology.

[34] Jiang ZH, Aierken A, Wu TT, Zheng YY, Ma YT, Xie X (2023). Rate pressure product as a novel predictor of long-term adverse outcomes in patients after percutaneous coronary intervention: a retrospective cohort study. BMJ Open.

[35] La Rovere MT, Pinna GD, Raczak G (2008). Baroreflex sensitivity: measurement and clinical implications. Annals of Noninvasive Electrocardiology : the Official Journal of the International Society for Holter and Noninvasive Electrocardiology, Inc.

[36] Sykora M, Diedler J, Rupp A, Turcani P, Rocco A, Steiner T (2008). Impaired baroreflex sensitivity predicts outcome of acute intracerebral hemorrhage. Critical Care Medicine.

[37] Sykora M, Diedler J, Turcani P, Hacke W, Steiner T (2009). Baroreflex: a new therapeutic target in human stroke?. Stroke.

[38] Hamann GF, Strittmatter M, Hoffmann KH, Holzer G, Stoll M, Keshevar T (1995). Pattern of elevation of urine catecholamines in intracerebral haemorrhage. Acta Neurochirurgica.

[39] Kalita J, Misra UK, Kumar B (2013). Is β-blocker (atenolol) a preferred antihypertensive in acute intracerebral hemorrhage?. Neurological Sciences.

[40] Sansing LH, Messe SR, Cucchiara BL, Lyden PD, Kasner SE (2011). Anti-adrenergic medications and edema development after intracerebral hemorrhage. Neurocritical Care.

[41] Shoup JP, Winkler J, Czap A, Staff I, Fortunato G, McCullough LD (2014). β-Blockers associated with no class-specific survival benefit in acute intracerebral hemorrhage. Journal of the Neurological Sciences.

[42] Balla HZ, Cao Y, Ström JO (2021). Effect of Beta-Blockers on Stroke Outcome: A Meta-Analysis. Clinical Epidemiology.

[43] Wang L, Ma L, Ren C, Zhao W, Ji X, Liu Z (2024). Stroke-heart syndrome: current progress and future outlook. Journal of Neurology.

[44] Ma S, Li J, Kong Q, Xu Z, Wu H, Jin Y (2025). Impact of Acute Myocardial Injury on Short- and Long-Term Outcomes in Patients With Primary Intracerebral Hemorrhage. Journal of the American Heart Association.

[45] Ishiguchi H, Huang B, El-Bouri WK, Dawson J, Lip GYH, Abdul-Rahim AH (2024). Incidence and Outcomes of Patients With Early Cardiac Complications After Intracerebral Hemorrhage: A Report From VISTA. Stroke.

[46] Hoad KL, Jones H, Miller G, Abdul-Rahim AH, Lip GY, Buckley BJ (2025). Stroke-heart syndrome: Incidence and clinical outcomes of cardiac complications following intracerebral haemorrhage. European Stroke Journal.

