# Supplementary material for: Early Cardiac Workload and Long-Term Prognosis After Intracerebral Hemorrhage: Insights from a Large Multicenter Cohort
Source: Rev Cardiovasc Med. 2026 Jun 17;27(6):49651. doi: 10.31083/RCM49651 (PMC13339233; doi:10.31083/RCM49651)
Supplement: Supplementary file 1 [file 2153-8174-27-6-49651-s1.zip › Supplementary Table 1.docx]

Supplementary Table 1. Sensitivity analysis.

|  | ≤6 h (n=838) | | ≤12 h (n=1058) | | ≤24 h (n=1189) | |
| --- | --- | --- | --- | --- | --- | --- |
|  | OR/HR (95% CI) | *p*-value | OR/HR (95% CI) | *p*-value | OR/HR (95% CI) | *p*-value |
| **90-day unfavorable functional outcome, OR (95% CI)** | | | | | | |
| n (%) | 375 (44.7) |  | 474 (44.8) |  | 516 (43.4) |  |
| Model 1 | 1.179 (1.131–1.228) | <0.001 | 1.174 (1.132–1.217) | <0.001 | 1.171 (1.132–1.211) | <0.001 |
| Model 2 | 1.187 (1.139–1.238) | <0.001 | 1.185 (1.142–1.230) | <0.001 | 1.185 (1.144–1.227) | <0.001 |
| Model 3 | 1.077 (1.019–1.138) | 0.008 | 1.078 (1.027–1.131) | 0.002 | 1.078 (1.030–1.129) | 0.001 |
| Model 4 | 1.086 (1.026–1.149) | 0.004 | 1.087 (1.034–1.142) | 0.001 | 1.088 (1.037–1.140) | 0.001 |
| **1-year unfavorable functional outcome, OR (95% CI)** | | | | | | |
| n (%) | 305 (36.4) |  | 389 (36.8) |  | 426 (35.8) |  |
| Model 1 | 1.162 (1.117–1.209) | <0.001 | 1.162 (1.122–1.203) | <0.001 | 1.156 (1.119–1.195) | <0.001 |
| Model 2 | 1.175 (1.128–1.225) | <0.001 | 1.179 (1.137–1.223) | <0.001 | 1.177 (1.136–1.218) | <0.001 |
| Model 3 | 1.059 (1.004–1.119) | 0.037 | 1.065 (1.015–1.118) | 0.010 | 1.068 (1.021–1.118) | 0.005 |
| Model 4 | 1.071 (1.012–1.132) | 0.017 | 1.078 (1.026–1.132) | 0.003 | 1.081 (1.031–1.133) | 0.001 |
| **90-day all-cause mortality, HR (95% CI)** | | | | | | |
| n (%) | 197 (23.5) |  | 244 (23.1) |  | 261 (22.0) |  |
| Model 1 | 1.143 (1.111–1.175) | <0.001 | 1.151 (1.123–1.180) | <0.001 | 1.151 (1.124–1.180) | <0.001 |
| Model 2 | 1.143 (1.111–1.176) | <0.001 | 1.153 (1.124–1.183) | <0.001 | 1.155 (1.127–1.184) | <0.001 |
| Model 3 | 1.027 (0.995–1.060) | 0.102 | 1.034 (1.006–1.064) | 0.019 | 1.035 (1.008–1.064) | 0.011 |
| Model 4 | 1.037 (1.005–1.071) | 0.025 | 1.041 (1.012–1.070) | 0.005 | 1.044 (1.016–1.072) | 0.002 |
| **1-year all-cause mortality, HR (95% CI)** | | | | | | |
| n (%) | 223 (26.6) |  | 284 (26.8) |  | 308 (25.9) |  |
| Model 1 | 1.137 (1.107-1.168) | <0.001 | 1.140 (1.113–1.168) | <0.001 | 1.141 (1.114–1.168) | <0.001 |
| Model 2 | 1.141 (1.110–1.172) | <0.001 | 1.145 (1.117–1.173) | <0.001 | 1.148 (1.121–1.176) | <0.001 |
| Model 3 | 1.027 (0.996–1.058) | 0.090 | 1.032 (1.005–1.061) | 0.021 | 1.037 (1.011–1.064) | 0.006 |
| Model 4 | 1.036 (1.005–1.068) | 0.022 | 1.039 (1.012–1.068) | 0.005 | 1.045 (1.018–1.072) | <0.001 |

Model 1: Unadjusted;

Model 2: Adjusted for age and sex;

Model 3: Adjusted for variables in Model 2 plus smoking, alcohol intake, medical history (hypertension, diabetes mellitus, dyslipidemia, ischemic stroke, myocardial infarction), modified Rankin Scale score before onset, admission Glasgow Coma Scale, location of hematoma, hematoma volume, intraventricular extension.

Model 4: Adjusted for variables in Model 3 plus in-hospital medications (antihypertensive agents, antidiabetic agents, lipid-lowering agents).

Abbreviations: CI, confidence interval; HR, hazard ratio; OR, odds ratio.
